# Identification of sexual myths of university students in health-related departments and affecting factors

**DOI:** 10.1590/1806-9282.20240416

**Published:** 2024-09-02

**Authors:** Ebru İnan Kırmızıgül, Sümeyra Damsarsan, Gonca Karataş Baran, Didem Şimşek Küçükkelepçe, Zehra Gölbaşı

**Affiliations:** 1 Lokman Hekim University, Faculty of Health Sciences, Department of Midwifery – Ankara, Turkey.; 2 University of Health Sciences, Faculty of Health Sciences, Department of Midwifery – Ankara, Turkey.; 3 Etlik Zübeyde Hanım Gynecology Training and Research Hospital – Ankara, Turkey.; 4 Lokman Hekim University, Faculty of Health Sciences, Department of Nursing – Ankara, Turkey.

**Keywords:** Belief, Health occupations students, Sexuality, Sexual health

## Abstract

**AIM:**

The research aimed to determine the attitudes of students studying in health-related departments toward sexual myths and the factors affecting them.

**METHODS:**

The study is descriptive research involving 287 students enrolled in health-related departments. The data were collected using a "Descriptive Information Form" and the "Sexual Myths Scale (SMS)" and analyzed using the SPSS 22.0 software package. The SPSS 22.0 package program was used to evaluate the data. In statistical analysis, Spearman correlation analysis was employed to determine the relationship between continuous variables and the SMS score, and the statistical significance level was accepted as p<0.05.

**RESULTS:**

The total score was found to be 53.57±17.54 (min: 28.00 to max: 140.00), reflecting a moderate level. There was a statistically significant difference between the total score of SMS according to gender, family type, maternal employment status, and paternal education level (p<0.05). It was also determined that male students, students whose mothers were unemployed, who lived in extended families, and whose fathers had low education had lower SMS scores.

**CONCLUSION:**

Despite students studying in health-related departments and receiving relevant courses, their level of sexual myths remains at a moderate level, indicating the presence of knowledge gaps and misconceptions in the subject matter. Therefore, it is crucial to implement comprehensive education and counseling services on reproductive and sexual health for all university students.

## INTRODUCTION

It encompasses sexual orientation, eroticism, intimacy, and reproduction, with gender playing a central role and significantly influencing an individual's quality of life^1^. Perspectives on sexuality are influenced by multiple factors, including personal meanings, beliefs, attitudes, values, and experiences with sexual roles and relationships, as well as the broader social structure and culture in which individuals reside^2^. The influence of social and cultural structures contributes to the formation of beliefs about sexuality that may lack truth but are accepted nonetheless. Although lacking scientific validity, these beliefs and information permeate society as integral components of the culture, shaped and mythologized by the collective imagination of individuals. Frequently, these beliefs manifest as exaggerated notions and thoughts regarding sexuality, commonly known as sexual myths^3^. Such sexual myths hinder individuals from openly expressing and discussing matters related to sexuality^4^. It is acknowledged that the university education period, during which young individuals socialize, develop their individual identities, and engage in sexual activity, has a significant impact on sexual myths and attitudes^5^. In particular, the perspectives of students studying in health-related fields regarding sexuality hold great importance. Given that sexuality is influenced by various factors throughout the lifespan, sexual health services are recognized as vital components of general healthcare and health promotion programs. However, research indicates that, despite having adequate knowledge, students often refrain from discussing patients’ sexual histories due to concerns regarding the reactions of patients, their families, and healthcare professionals^6,7^. Numerous studies have highlighted that nursing students who hold moderate levels of sexual myths encounter increased perceived barriers when it comes to evaluating and providing sexual care, leading to difficulties in delivering comprehensive care^6,8,9^. Given that these students are future healthcare professionals, their sexual myths carry substantial significance as they directly impact the effective provision of care and the overall health of society. Therefore, this study was conducted to examine the attitudes of students pursuing careers in healthcare toward sexual myths and identify the factors influencing these attitudes.

## METHODS

Study setting: Between February and June 2022, this descriptive research was conducted among students enrolled in health-related departments at a foundation university.

Population and sample: The target population consisted of 2,167 students studying various health-related disciplines at a private university in Turkey during the Spring Term of the academic year 2021–2022. The sample size was determined using the formula established by Salant and Don^10^. Using the sampling formula, the required sample size was calculated as n=221 with a 95% confidence interval and±5% sampling error for this population, which is not homogenous. In the post hoc power analysis, comparing the scale score averages according to gender, one of the factors affecting the Sexual Myths Scale (SMS), the effect size was found to be 1.167 and the power of the study was determined to be 0.99.

Ethical approval: This study was performed in line with the principles of the Declaration of Helsinki. Approval was granted by the Ethics Committee (Date 15. 03.2022/No: 2022035). Before the study, written consent was obtained indicating that they agreed to participate in the study. It was specifically stated to the students that this study did not have any grade impact.

Data collection and data collection tools: For this study, the researchers collected the data using a face-to-face interview technique when the students were not attending classes. Two data collection tools were utilized: the "Introductory Information Form" and the "SMS."

Descriptive information form: Developed by the researchers based on the existing literature, this form consists of 15 questions to evaluate the students’ sociodemographic characteristics, education-related attributes, and perspectives on sexuality^6,8,9^. The student's level of knowledge about sexuality and their comfort level when discussing sexuality is assessed through questions where they subjectively rate themselves on a scale ranging from 0 to 10 (0: Strongly Disagree to 10: Strongly Agree).

The SMS: Created by Golbası et al. in 2016, the SMS is a 5-point Likert-type scale, with responses such as "completely agree" (5), "somewhat agree" (4), "not sure" (3), "disagree" (2), and "completely disagree" (1). The minimum and maximum scores on the scale are 28 and 140. The scale measures the extent to which individuals believe in sexual myths, with a higher score indicating a higher probability of belief^11^. The Cronbach's alpha coefficient for the SMS was determined to be 0.91, and the coefficient for the test–retest reliability study was found to be 0.814. In this study, Cronbach's alpha value for the scale was calculated to be 0.93.

### Data analysis

The SPSS 22.0 package program was employed for data analysis. The conformity of the variables to the normal distribution was evaluated with the Shapiro-Wilk test. Percentages, mean±standard deviation, and median (min–max) were used to represent descriptive statistics. The distribution of scale scores in independent groups was evaluated with the Mann-Whitney U test and the Kruskal-Wallis test. Median (min–max)/Mean rank was used for descriptive data that were not normally distributed. The relationship between continuous variables and scale scores was evaluated using Spearman's correlation analysis. The statistical significance level was accepted p<0.05.

## RESULTS

For students with a mean age of 20.59±1.58 years, 84.7% of participants expressed their belief that there should be a course on sexuality. The average level of knowledge about sexuality was found to be 7.01±1.91. The average comfort level score was 7.60 ±2.42 when discussing sexuality within the family and 4.37±2.22 when talking about sexuality among friends. Table 1 shows the data related to the level of knowledge about sexuality and feeling uncomfortable while talking about sexuality.

**Table 1:** Level of knowledge about sexuality and feeling comfortable when talking about sexuality.

		N (287)	% (100.0)
Should there be a sexual health course?			
	Yes	243	84.7
	No	14	4.9
	No idea	30	10.5
Level of knowledge about sexuality and feeling uncomfortable talking about sexuality		Mean ±SD	Med (Min–Max)
Sexual knowledge level		7.01±1.91	7 (1–10)
Comfort level when talking about sexuality in the family		4.37±2.22	4 (1–10)
Comfort level when talking with friends about sexuality (same sex)		7.60±2.42	8 (1–10)

1 Mean: average; SD: standard deviation; Med: median; Min: minimum; Max: maximum.

Characteristics related to the mean scores of the SMS and subscales are as follows: SMS total score 53.57±17.54, 51.00 (28.00–140.00); Sexual orientation sub-dimension scale score 12.61±4.93, 12.00 (5.00–25.00); Gender subscale score 9.56±4.22, 8.00 (6.00–30.00); Age and Sexuality subscale score 7.45±3.30, 7.00 (4.00–20.00); Sexual Behavior subscale score 4.67±2.26, 4.00 (3.00–15.00); Masturbation subscale score 4.32±2.15, 4.00 (2.00–10.00); Sexual Violence subscale score 6.13±2.56, 5.00 (4.00–20.00); Sexual Intercourse subscale score 4.50±2.05, 4.00 (2.00–10.00); and Sexual satisfaction subscale score 4.33±1.87, 4.00 (2.00–10.00). Table 2 shows the characteristics related to the mean scores of the SMS and subscales. In addition, Table 2 shows the correlation findings of the SMS. No statistically significant correlation was found between the SMS score and age, level of knowledge about sexual issues, or comfort level when talking about sexual issues in the family (p>0.05). There was a negative, weak, statistically significant correlation between the level of comfort when talking about sexual issues with friends (p<0.05). As the SMS score increased, the level of comfort when talking about sexual issues with friends decreased (Table 2).

**Table 2:** Total and subscale score averages of the Sexual Myths Scale and correlation findings of the Sexual Myths Scale score and some variables.

Sexual Myths Scale		Mean±SD	Med (Min–Max)
Sexual Myths Scale total score		53.57±17.54	51.00 (28.00–140.00)
Sub-dimensions			
	Sexual orientation	12.61±4.93	12.00 (5.00–25.00)
	Gender	9.56±4.22	8.00 (6.00–30.00)
	Age and sexuality	7.45±3.30	7.00 (4.00–20.00)
	Sexual behavior	4.67±2.26	4.00 (3.00–15.00)
	Masturbation	4.32±2.15	4.00 (2.00–10.00)
	Sexual violence	6.13±2.56	5.00 (4.00–20.00)
	Sexual intercourse	4.50±2.05	4.00 (2.00–10.00)
	Sexual satisfaction	4.33±1.87	4.00 (2.00–10.00)
Some variables		Sexual Myths Scale	
Age		ρ=–0.026	p=0.662
Level of knowledge on sexuality		ρ=–0.078	p=0.190
Comfort level when talking about sexuality in the family		ρ=–0.101	p=0.088
Comfort level when talking with friends about sexuality (same-sex)		ρ=-0.162	p=0.006

SD: standard deviation; ρ: Spearman's rho correlation coefficient.

Table 3 shows the relationship between the SMS and some variables. The SMS score of male students was found to be statistically significantly higher than that of female students, and those in extended family structures were statistically significantly higher than those in nuclear family structures. While there was no difference between the groups in terms of marital status, maternal education, paternal employment status, place of residence, and class (p>0.05), the SMS score was statistically significantly higher in those whose mothers were unemployed than those whose mothers were employed; in those whose fathers were secondary school graduates than those whose fathers were primary school graduates; in those whose fathers were unemployed than those whose fathers were employed; and in those who were medical faculty students than midwifery students (p<0.05).

**Table 3:** The relationship of Sexual Myths Scale with some variables.

Sociodemographic characteristics		Sexual Myths Scale		Analysis
		n	Med (min–max)/M. Rank	
Gender				
	Male	46	69.50 (40–104)/219.21	z=-6.709 p=0.000
	Female	241	49.00 (28–140)/129.65	
Marital status				
	Married	4	52.50 (33–80)/149.38	z=-0.130 p=0.896
	Single	283	51.00 (28–140)/143.92	
Family type				
	Nuclear	246	49.00 (28–140)/137.43	z=-3.284 p=0.001
	Extended	41	60.00 (28–116)/183.40	
Maternal education				
	Illiterate	2	59.50 (49–70)/186.50	χ^2^=2.411 df=5 p=0.790
	Literate	5	54.00 (28–96)/136.00	
	Primary school	61	54.00 (30–140)/150.39	
	Secondary school	50	54.00 (29–91)/151.98	
	High school	85	49.00 (28–106)/134.96	
	University and above	84	50.00 (28–116)/143.21	
Maternal employment				
	Yes	97	47.00 (28–96)/1236.58	z=-2.542 p=0.011
	No	190	54.00 (28–140)/152.89	
Paternal education				
	Primary school	25	43.00 (28–80)/114.96	χ^2^=8.539 df=3 p=0.036 a–b
	Secondary school	40	58.00 (30–104)/169.95	
	High school	85	48.00 (28–140)/133.94	
	University and above	137	52.00 (28–116)/147.97	
Paternal employment				
	Yes	246	50.00 (28–140)/138.91	z=-2.543 p=0.011
	No	41	62.00 (28–116)/174.51	
Permanent place of residence				
	City	228	50.00 (28–140)/142.21	χ^2^=0.620 df=2 p=0.734
	District	51	54.00 (28–93)/149.52	
	Town/village	8	56.50 (28–89)/159.75	
Department				
	Faculty of Medicine	80	59.00 (33–116)/176,62	χ^2^=21.783 df=7 p=0.003 (a–d)
	Nutrition and Dietetics	10	59.00 (28–73)/173.30	
	Speech and Language Therapy	14	46.00 (32–75)/124.14	
	Midwifery	157	49.00 (28–140)/129.71	
	Ergotherapy	3	56.00 (39–62)/148.33	
	Physiotherapy and Rehabilitation	8	54.50 (31–72)/143.38	
	Nursing	12	38.50 (28–89)/106.17	
	Vocational School Departments	3	59.00 (34–90)/165.83	
Year				
	1	84	49.00 (28–106)/136.08	χ^2^=6.097 df=3 p=0.107
	2	108	54.00 (28–140)/158.19	
	3	70	51.00 (28–96)/140.00	
	4	25	48.00 (28–73)/120.48	

M. Rank: mean rank; ρ: Spearman's rho correlation coefficient; z: Mann-Whitney U test; χ^2^: Kruskal-Wallis test. Statistically significant values are indicated in bold.

## DISCUSSION

In Turkey, sexuality is seen as a taboo due to cultural and religious reasons, and topics related to sexuality are avoided from being discussed^11^. However, it is generally observed that young people do not receive science-based education about sexual health throughout their education life. It is observed that only students studying in health-related departments take courses on sexual health^12^. For this reason, students studying in health-related departments are considered to have very good knowledge of sexual health. Additionally, they are thought not to have many sexual myths^12^. Contrary to what is thought, in the study of Junior et al., it was reported that adolescents’ knowledge about STIs was not sufficient^13^. Lack of knowledge about sexuality may cause sexual myths to be more common in young people. Lack of information also paves the way for reproductive and sexual health problems. Studies report that the sexuality of women with gynecological problems is affected to certain degrees^14,15^. The relationship between sexuality, sexual health, and sexual myths can be thought of as a circle that affects each other. According to the findings of this study, unfortunately, the sexual health knowledge and sexual myths of students studying in health-related departments are not at the desired level. For this reason, determining the attitudes and influencing factors of university students studying in health-related departments regarding sexual myths, which will shape the health of the society, is important for raising and educating healthy individuals in the society.

Awareness, education, and maintenance of sexual health are crucial factors in promoting the well-being of individuals concerning sexuality and sexual health^16^. It is noteworthy that 84.7% of the students who took part in our study expressed the need for a comprehensive sexuality course to be included in their education curriculum. Furthermore, when asked to rate their average level of knowledge on sexual issues on a scale from 1 to 10, the students reported an average score of 7.01±1.91, indicating a perceived high and satisfactory level of knowledge. Notably, 65.54 and 79.3% of university students considered their knowledge about sexuality to be sufficient in a study conducted by Örüklü et al. and Doğan et al., respectively^17,18^. As in other societies worldwide, sexual behaviors and attitudes in Turkish society are significantly influenced by religious regulations, prejudices, taboos, customs, and traditions^19^. Specifically, within the context of Turkish society, individuals tend to feel more comfortable discussing sexuality with their peers than with their families^20^. Our study's results support this notion, as the comfort level score of the participating students was 4.37±2.22 when discussing sexuality within the family, whereas it increased to 7.60±2.42 when talking with friends.

Numerous factors, such as family dynamics, cultural influences, physiological aspects, religious beliefs, and psychological conditions, contribute significantly to the development of an individual's sexual attitude^21^. Recognizing the significance of understanding personal sexual misconceptions, it is crucial for students pursuing health-related disciplines to explore their own sexual beliefs before engaging in the assessment of others’ sexuality and delivering effective sexual counseling^6^. In our research, the average score for sexual myths was determined to be 53.57±17.54. A study by Örüklü et al. examining the perspectives of university students regarding sexual myths reported a mean score of 61.02±19.10^17^. Similarly, Öz et al. found a score of 56.77±17.8 among nursing students in a comparable study, while Evcili and Demirel reported a score of 76.43±17.09 among nursing and midwifery students^6,19^. Along with existing literature, our study reveals that students generally hold moderate myths about sexuality. Research indicates that students with moderate levels of sexual myths face increased perceived barriers when addressing sexuality in caregiving and encounter difficulties in providing appropriate care^6,8,9^.

Notably, the perspective on sexuality is influenced by various factors, including gender^17^. A study conducted among Turkish students demonstrated that sexual myths were more prevalent among male students^21^. Likewise, our study revealed higher levels of sexual myths among male students compared with their female counterparts. These differences in beliefs and attitudes between genders may be attributed to the distinct societal values associated with male and female sexuality in Turkish culture.

In our study, marital status did not significantly influence the score of sexual myths. However, previous studies conducted with different populations have reported higher sexual myth scores among married individuals compared with those who are single. This difference is believed to stem from variations in sexual knowledge and experiences^22,23^.

The nuclear family structure is considered a significant reflection of modernization^24^. In our study, we speculate that the lower sexual myths score among students from nuclear family structures is attributed to having a more modern family setup.

Although there was no notable difference between the educational level of the students’ mothers and their sexual myths scores, those whose mothers were illiterate exhibited the highest sexual myths score, suggesting that the maternal education level has an impact on the presence of sexual myths in their children.

According to the results of the Household Labor Force Survey, the labor force participation rate for individuals aged 15 years and over was reported as 51.4% in 2021, with rates of 32.8% for women and 70.3% for men^25^, which is consistent with our study. Considering that the participation of both men and women in the labor force in Turkey improves the socio-economic status of families, it can be inferred that individuals whose parents are employed have lower sexual myth scores compared with those whose parents are unemployed. Furthermore, medical faculty students had higher SMS scores than those in the midwifery department. These findings suggest that the level of belief in sexual myths among students studying health-related disciplines may vary across different departments.

Although there was no statistically significant difference between the class levels of the students and their sexual myths scores, it was observed that fourth-year students had lower sexual myths scores compared with lower-class students. This could be attributed to the lack of completion of courses related to sexual and reproductive health until the fourth year, which may contribute to a decrease in sexual myth scores.

### Limitations of the research

As the research was conducted with students at a university located in the capital of Turkey and this university accepts students from many different provinces of the country, it is thought to be a sample close to national representation. However, as the study was conducted at a single university, its generalizability to the whole country is limited.

## CONCLUSION AND RECOMMENDATIONS

Despite studying in a health-related department and taking relevant courses, the moderate level of sexual myths among the participants indicates that, contrary to popular belief, there are still knowledge gaps and misconceptions regarding sexuality among students. Considering these findings, it is recommended to provide comprehensive reproductive health and sexual health education, particularly targeting university students enrolled in health-related departments, and expand the content of these courses to address common misconceptions about sexuality.

## ETHICAL APPROVAL

The study was performed in accordance with the ethical standards of the institutional and/or national research committee and with the 1964 Helsinki Declaration and its later amendments and the Good Clinical Practice Guidelines. Before starting the study, ethics committee approval numbered 2022035 (2022/44) was obtained from the Ethics Committee of Lokman Hekim University. Informed consent was obtained from all individual participants included in the study.

## Data Availability

The authors confirm that the data supporting the findings of this study are available within the article.

## References

[1] Ventriglio A, Bhugra D. (2019). Sexuality in the 21st century: sexual fluidity. East Asian Arch Psychiatry.

[2] Dolapoğlu N, Taşkın M, Altunöz S. (2023). The relationship between pregnant women and their spouses’ belief in sexual myths during pregnancy, relationship satisfaction and sexual satisfaction. J Health Scie Med.

[3] Turkish Language Association (2023). Current Turkish dictionary. Https://Sozluk.Gov.Tr/.

[4] (2021). Association of Sexual Health Institute. Sexual myths. https://www.cised.org.tr/icerik/146/cinsel-mitler.

[5] Davul ÖE, Yazıcı AE. (2019). The effect of university life on sexual myths and attitudes. Cukurova Med J.

[6] Öz HG, Sözer GA, Yangın HB (2020). Nursing students’ beliefs in sexual myths and affecting factors. Ordu Uni J Nurs Stud.

[7] Kong SK, Wu LH, Loke AY. (2009). Nursing students’ knowledge, attitude and readiness to work for clients with sexual health concerns. J Clin Nurs.

[8] Aşcı Ö, Gökdemir F. (2021). Sexual myths and attitudes towards evaluating patient sexuality in nursing students.

[9] Akalin A, Ozkan B. (2021). Sexual myths and attitudes regarding sexuality of nursing students: a mixed method study. Perspect Psychiatr Care.

[10] Salant P, Don AD. (1994). How to conduct your own survey.

[11] Golbası Z, Evcılı F, Eroglu K., Bircan H. (2016). Sexual myths scale: development, validity and reliability in Turkey. Sex Disabil.

[12] Uğurlu M, Karahan N. (2022). Sexual health knowledge and influencing factors among health science students at a state university in Turkey. Eur J Contracept Reprod Health Care.

[13] Soares JM, Oliveira HMC, Luquetti CM, Zuchelo LTS, Arruda Veiga EC, Raimundo JZ (2022). Adolescents’ knowledge of HPV and sexually transmitted infections at public high schools in São Paulo: a cross-sectional study. Clinics (Sao Paulo).

[14] Firmino Murgel AC, Santos Simões R, Maciel GAR, Soares JM, Baracat EC. (2019). Sexual dysfunction in women with polycystic ovary syndrome: systematic review and meta-analysis. J Sex Med.

[15] Silva JS, Fonseca AM, Bagnoli VR, Cavalcanti AL, Soares JM, Baracat EC. (2010). Sexuality in women with polycystic ovary syndrome: a pilot study. Einstein (Sao Paulo).

[16] Gürsoy DE, Gençalp NS. (2010). Importance of sexual health education. J Soc Pol Stud.

[17] Örüklü C, Dağcı DG, Çakmak S. (2021). University students’ perspective on sexual myths and associated factors. Istanbul Gelisim Uni J Health Sci.

[18] Doğan M, Solmaz Y, Eycan Ö, Abdan M, Doğan M, Güder P (2022). Attitudes and views of university students regarding sexual myths/sexual assault myths. Bull Leg Med.

[19] Evcili F, Demirel G. (2018). Sexual myths of midwifery and nursing students and their attitude regarding the assessment of sexual health. J Hum Sci.

[20] Sümer ZH. (2015). Gender, religiosity, sexual activity, sexual knowledge, and attitudes toward controversial aspects of sexuality. J Relig Health.

[21] Vefikuluçay YD, Güner ET, Uzel A, Değirmenci F, Buldum A, Aksu A (2020). Determination of nursing students’ sexual myths. Arch Health Sci Res.

[22] Aker S, Böke Ö. (2016). The effect of education on the sexual beliefs of family physicians. Int J Sexual Health.

[23] Kahraman S, Kaya ME, Erez I. (2021). Examining the relationship between perfectionism and sexual myths in adults. Cyprus Turk J Psychiatry Psychol.

[24] Karsli E. (2019). The dissolved family structure and the reconstruction of women in the modernization process. Int J Law Soc Sci Stud.

[25] Turkish Statistical Institute (2023). Labor statistics. https://data.tuik.gov.tr/Bulten/Index?p=Isgucu-Istatistikleri-2023-53521.

